# Supplementary material for: Distinct homeostatic modulations stabilize reduced postsynaptic receptivity in response to presynaptic DLK signaling
Source: Nat Commun. 2018 May 10;9:1856. doi: 10.1038/s41467-018-04270-0 (PMC5945772; doi:10.1038/s41467-018-04270-0)
Supplement: Supplementary file 1 — Supplementary Information [file 41467_2018_4270_MOESM1_ESM.pdf]

Distinct homeostatic modulations stabilize reduced postsynaptic receptivity in response to presynaptic DLK signaling

Goel and Dickman

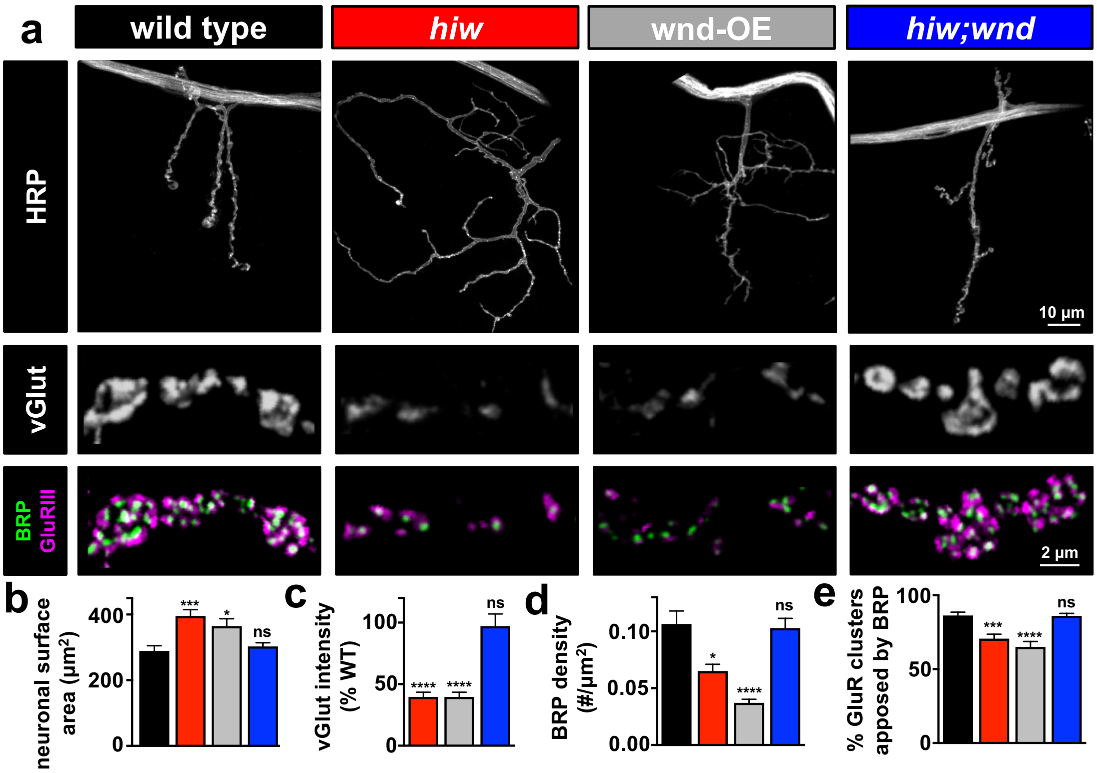

a
wild type
hiw
wnd-OE
hiw;wnd
HRP
10 µm
vGlut
BRP
GluRIII
2 µm
b
neuronal surface area (µm²)
400
200
0
***
*
ns
c
vGlut intensity (% WT)
100
50
0
****
****
ns
d
BRP density (#/µm²)
0.10
0.05
0.00
*
****
ns
e
% GluR clusters apposed by BRP
100
50
0
***
****
ns

Supplementary Fig 1: Presynaptic Wnd signaling leads to increased synaptic growth and reductions in vGlut levels and BRP density. (a) Representative images of third-instar larval muscle 4 NMJs of wild type (w^{1118}), *highwire* mutants (*hiw*: $hiw^{\Delta N}$), neuronal overexpression of *wnd* (wnd-OE: *c380-Gal4/+;UAS-wnd/+*) and *hiw;wnd* double mutants (*hiw;wnd:* $hiw^{\Delta N}$;wnd^{3}/wnd^{Df}) immunostained with antibodies that recognize the neuronal membrane marker HRP (white). *Lower panels*: Representative images of individual boutons immunostained with antibodies that recognize the synaptic vesicle marker vGlut (vesicular glutamate transporter), the presynaptic active zone scaffold Bruchpilot (BRP) and postsynaptic glutamate receptor subunit GluRIII. Quantification of neuronal membrane surface area **(b)**, average intensity of each individual vGlut puncta **(c)**, BRP puncta density **(d)**, and BRP-GluRIII apposition (percentage of glutamate receptor puncta with an apposed BRP puncta) **(e)**. Activation of Wnd signaling leads to an increase in synaptic growth, a reduction in presynaptic proteins namely vGlut and Brp, and a mild but significant defect in active zone-receptor apposition. Error bars indicate ±SEM. One-way analysis of variance (ANOVA) test was performed, followed by a Tukey's multiple-comparison test. **p 0.01; ***p 0.001; ****p=0.0001, ns=not significant, p>0.05.

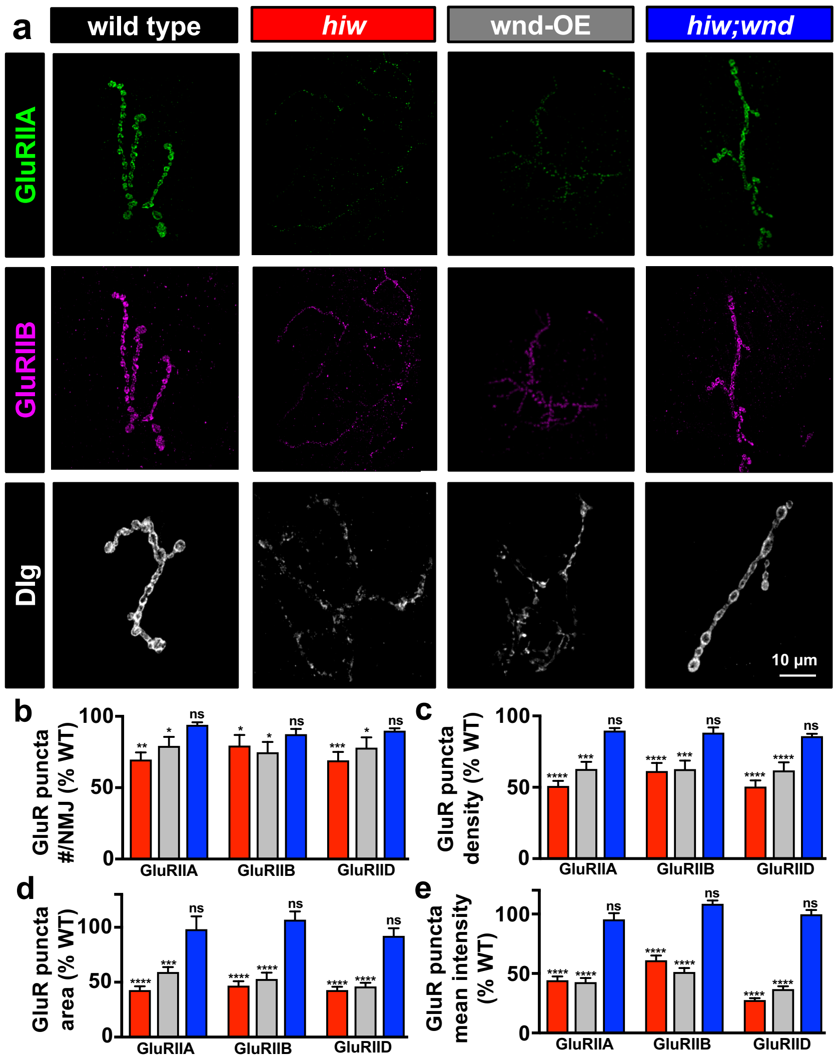

a
wild type
hiw
wnd-OE
hiw;wnd
GluRIIA
GluRIIB
Dlg
10 µm
b
GluR puncta #/NMJ (% WT)
GluRIIA
GluRIIB
GluRIID
c
GluR puncta density (% WT)
d
GluR puncta area (% WT)
e
GluR puncta mean intensity (% WT)
ns

Supplementary Fig 2: Presynaptic Wnd signaling diminishes postsynaptic glutamate receptor levels and Dlg. (a) Representative images of muscle 4 NMJs of wild type (*w^{1118}*), *highwire* mutants (*hiw*: *$hiw^{\Delta N}$*), neuronal overexpression of *wnd* (wnd-OE: *c380-Gal4/+;UAS-wnd/+*) and *hiw;wnd* double mutants (*hiw;wnd: $hiw^{\Delta N}$;wnd^{3}/wnd^{Df}*) immunostained with antibodies that recognize the postsynaptic glutamate receptor subunits GluRIIA, GluRIIB, and the postsynaptic scaffold (Dlg). Note the reduced intensity levels of the labeled postsynaptic proteins across the entire NMJ in *hiw* and wnd-OE, which are suppressed in *hiw;wnd* mutants. A reduction in the total number **(b)** and density **(c)** of receptor puncta (GluRIIA, GluRIIB, and GluRIID) is observed in *hiw* and wnd-OE. Quantification of each individual glutamate receptor puncta area **(d)** and mean intensity **(e)** demonstrates a significant reduction in the size and intensity of GluR puncta. Error bars indicate ±SEM. One-way analysis of variance (ANOVA) test was performed, followed by a Tukey's multiple-comparison test. *p=0.05; **p=0.01; ***p=0.001; ****p=0.0001, ns=not significant, p>0.05.

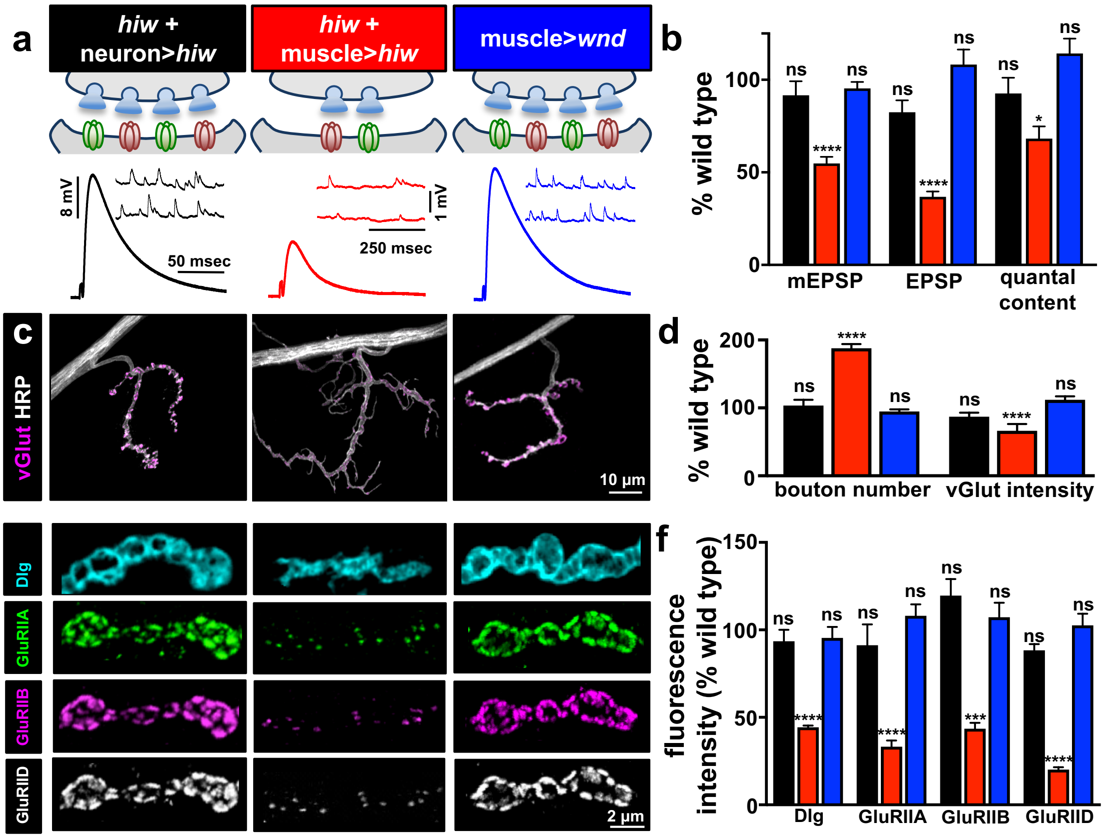
a
hiw + neuron>hiw
hiw + muscle>hiw
muscle>wnd
8 mV
50 msec
1 mV
250 msec
b
% wild type
mEPSP
EPSP
quantal content
ns
****
*
c
vGlut HRP
10 µm
d
% wild type
bouton number
vGlut intensity
Dlg
GluRIIA
GluRIIB
GluRIID
2 µm
f
fluorescence intensity (% wild type)
***

Supplementary Fig 3: Neuronal functions of *hiw* and *wnd* are necessary for proper synaptic structure and function. (a) Schematic and representative EPSP and mEPSP traces of *hiw* mutants with expression of *hiw* in motor neurons (*hiw*+neuron>*hiw*: *hiw*$^{\Delta N}$*;OK6-Gal4/UAS-hiw*) or muscle (*hiw*+muscle>*hiw*: *hiw*$^{\Delta N}$*;UAS-hiw*/+;*MHC-Gal4*/+), as well as *wnd* overexpression in the muscle (muscle>*wnd*: *w*^{1118}*;UAS-wnd/*+;*MHC-Gal4*/+). **(b)** Quantification of mEPSP, EPSP, and quantal content normalized to wild-type values, demonstrating that motor neuron expression of *hiw* rescues functional defects in *hiw* mutants, while muscle expression does not. Postsynaptic overexpression of *wnd* has no significant effect on synaptic physiology. **(c)** Representative images of muscle 4 NMJs of the indicated genotypes immunostained with antibodies that recognize the synaptic vesicle marker vGlut and the neuronal membrane marker HRP. **(d)** Quantification of bouton numbers and vGlut intensity levels normalized to wild-type levels. Note that the enhanced bouton numbers and reduced vGlut intensity levels characteristic of *hiw* mutants are rescued by presynaptic, but not postsynaptic, expression of *hiw*, while muscle overexpression of *wnd* does not significantly alter synaptic growth or structure. **(e)** Representative images of boutons in the indicated genotypes immunostained with antibodies against Dlg and GluRIIA, GluRIIB, and GluRIID subunits. **(f)** Quantification of Dlg, GluRIIA, GluRIIB and GluRIID fluorescence intensity levels normalized to wild type; only presynaptic *wnd* signaling impacts postsynaptic scaffolding and receptor levels. Error bars indicate ±SEM. One-way analysis of variance (ANOVA) test was performed, followed by a Tukey's multiple-comparison test. *p=0.05; ***p=0.001; ****p=0.0001, ns=not significant, p>0.05.

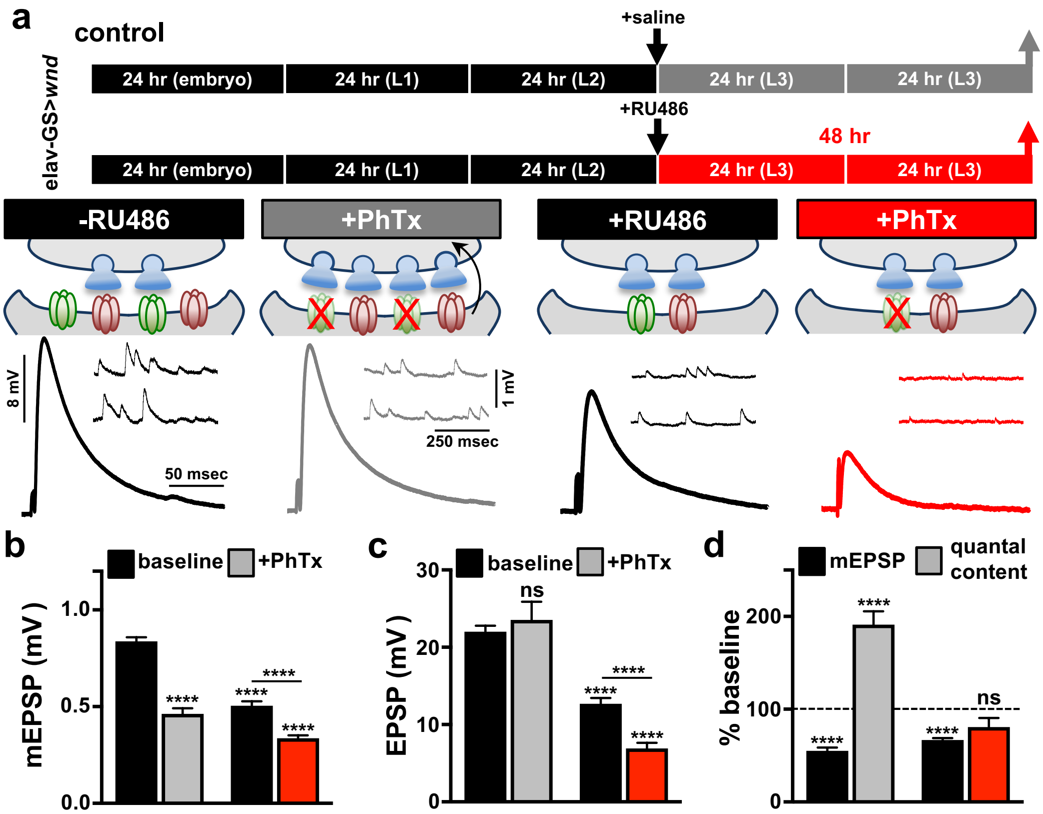
a
control
+saline
elav-GS>wnd
24 hr (embryo)
24 hr (L1)
24 hr (L2)
24 hr (L3)
24 hr (L3)
+RU486
48 hr
24 hr (embryo)
24 hr (L1)
24 hr (L2)
24 hr (L3)
24 hr (L3)
-RU486
+PhTx
+RU486
+PhTx
8 mV
50 msec
1 mV
250 msec
b
baseline
+PhTx
mEPSP (mV)
1.0
0.5
0.0
****
****
****
****
c
baseline
+PhTx
EPSP (mV)
30
20
10
0
ns
****
****
****
d
mEPSP
quantal content
% baseline
200
100
0
****
****
****
ns

Supplementary Fig 4: Presynaptic homeostatic potentiation is disrupted following acute activation of neuronal *wnd* signaling. (a) Schematic showing administration of RU486 to larvae at various time points to induce *wnd* expression (*elav-Geneswitch;UAS-wnd*). **(b)** Schematic and representative EPSP and mEPSP traces of recordings of larvae in the indicated conditions. Quantification of mEPSP amplitude **(c)** and EPSP amplitude **(d)** in the indicated conditions shows a reduction in mEPSP and EPSP amplitude after acute activation of *wnd* signaling, demonstrating that PHP fails to be expressed. **(e)** Quantification of mEPSP and quantal content values normalized to baseline (without PhTx application) in the same condition. No homeostatic increase in quantal content is observed in wnd-OE+RU486. Error bars indicate ±SEM. For statistical significance, an unpaired t-test was performed. ****p 0.0001; ns=not significant, p>0.05.

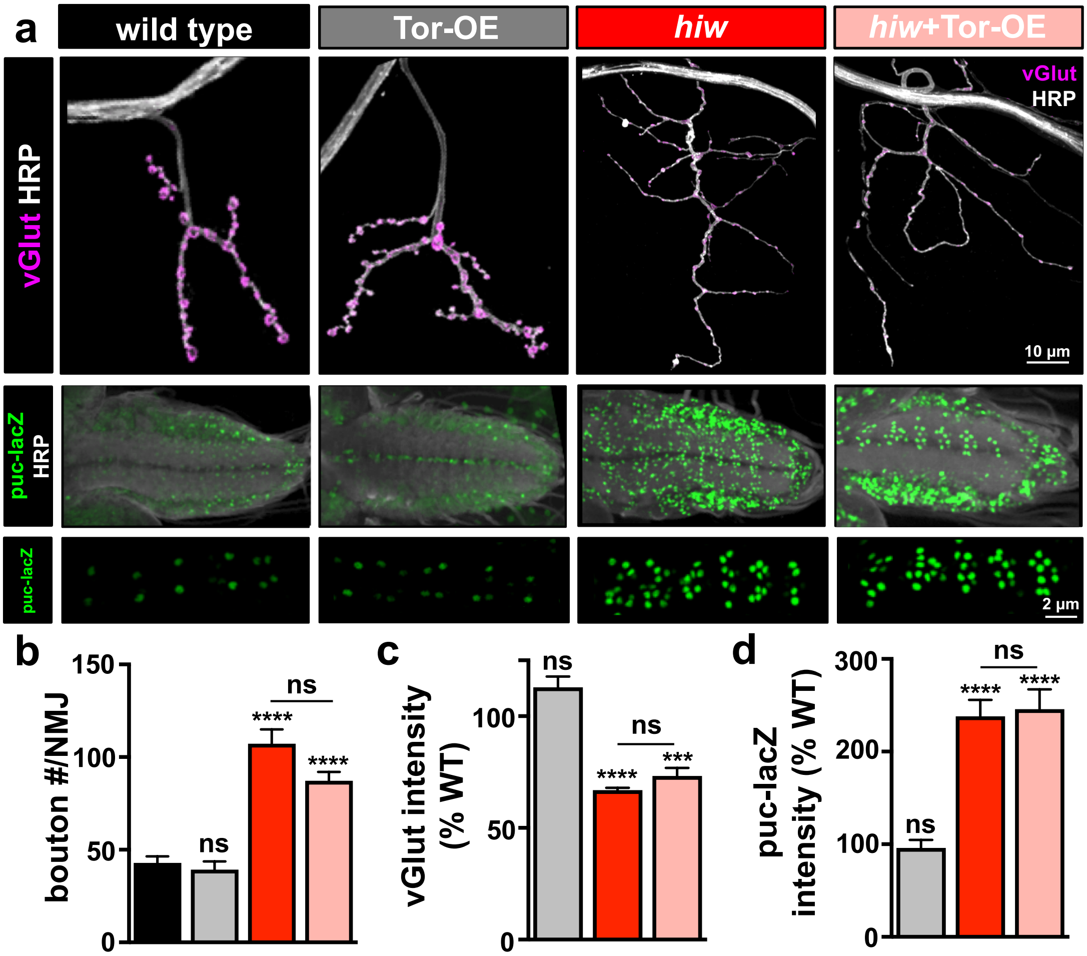
a
wild type
Tor-OE
hiw
hiw+Tor-OE
vGlut HRP
vGlut
HRP
10 µm
puc-lacZ HRP
puc-lacZ
2 µm
b
bouton #/NMJ
150
100
50
0
ns
****
****
ns
c
vGlut intensity (% WT)
100
50
0
ns
ns
****
***
d
puc-lacZ intensity (% WT)
300
200
100
0
ns
****
****
ns

Supplementary Fig 5: Postsynaptic Tor overexpression has no impact on presynaptic *wnd* signaling. (a) Representative images of muscle 4 NMJs in wild type, Tor-OE (Tor-OE: *w;MHC-Gal4/UAS-Tor*), *hiw* mutants, and muscle overexpression of Tor in *hiw* mutants (*hiw*+Tor-OE: *hiw*$^{\Delta N}$;*MHC-Gal4/UAS-Tor*) immunostained with anti-vGlut and anti-HRP. *Lower panels*: The puc-lacZ reporter was used to measure presynaptic Wnd signaling in wild type (*w;puc-lacZ/+*), Tor-OE (Tor-OE: *w;G14-Gal4/UAS-Tor;puc-lacZ/+*), *hiw* mutants (*hiw*$^{\Delta N}$*;puc-lacZ/+*), and muscle overexpression of Tor in *hiw* mutants (*hiw*+Tor-OE: *hiw*$^{\Delta N}$;*G14-Gal4/UAS-Tor;puc-lacZ/+*). lacZ expression in the nuclei of dorsal midline neurons in the ventral nerve cord is barely detectable in wild type and Tor-OE, but significantly elevated in both *hiw* and *hiw*+Tor-OE. Quantification of bouton numbers **(b)**, vGlut intensity **(c)**, and lacZ intensity **(d)** demonstrates that Tor-OE in *hiw* mutants has no significant impact on the changes in bouton numbers or vGlut/lacZ intensity characteristic of neuronal DLK signaling. Error bars indicate ±SEM. One-way analysis of variance (ANOVA) test was performed, followed by a Tukey's multiple-comparison test. ***p=0.001; ****p=0.0001; ns=not significant, p>0.05.

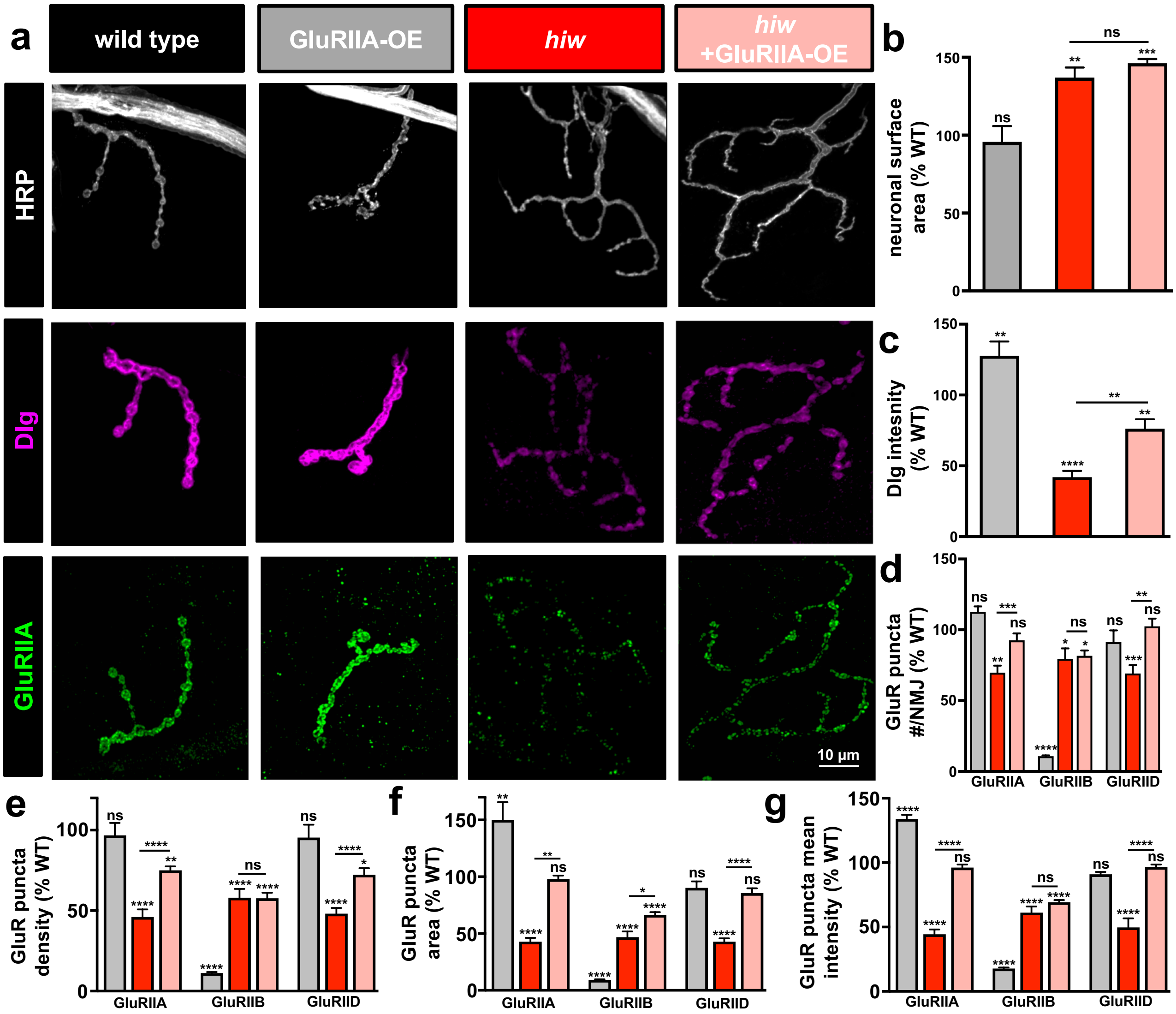

a
wild type
GluRIIA-OE
hiw
hiw +GluRIIA-OE
HRP
Dlg
GluRIIA
10 µm
b
neuronal surface area (% WT)
c
Dlg intesnity (% WT)
d
GluR puncta #/NMJ (% WT)
e
GluR puncta density (% WT)
f
GluR puncta area (% WT)
g
GluR puncta mean intensity (% WT)
GluRIIA
GluRIIB
GluRIID

Supplementary Fig 6: *GluRIIA* overexpression impacts glutamate receptor and Dlg levels in both wild type and *hiw* mutant NMJs. (a) Representative images of muscle 4 NMJs of wild-type, muscle overexpression of *GluRIIA* (GluRIIA-OE: *w;MHC-Gal4/UAS-dGluRIIA*), *hiw*-mutant, and *hiw*+GluRIIA-OE (*hiw*+GluRIIA-OE: $hiw^{\Delta N}$;*MHC-Gal4/UAS-dGluRIIA*) NMJs immunostained with anti-HRP, -Dlg, and -GluRIIA. GluRIIA-OE does not impact synaptic growth but enhances Dlg and GluRIIA puncta intensity levels. Quantification of neuronal membrane surface area **(b)**, Dlg intensity **(c)**, and GluRIIA puncta intensity **(d)** in the indicated genotypes. Quantification of GluR puncta density **(e)**, area **(f)**, and intensity **(g)** in the indicated genotypes. Error bars indicate ±SEM. One-way analysis of variance (ANOVA) test was performed, followed by a Tukey's multiple-comparison test. *p=0.05; **p=0.01; ***p=0.001; ****p=0.0001, ns=not significant, p>0.05.

Supplementary Table 1: Absolute values for normalized data and additional statistics. The figure and panel, genotype, and conditions used are noted (whether PhTx or RU486 was applied). For electrophysiological recordings, average values for mEPSP, EPSP, quantal content (QC), resting potential, input resistance, number of data samples (n), p values, and significance are shown. For confocal imaging analysis, average values for intensity levels of different synaptic markers and other relevant parameters such as numbers and area are shown. Standard error values are noted in parentheses. Rows highlighted in blue are the respective controls/baseline values for the particular experiment being referenced.

Figure	Genotype	mEPSP (mV)	EPSP (mV)	QC	mEPSP (Hz)	Resting Potential (mV)	Input Resistance (MΩ)	n	P value (significance) (mEPSP amp, EPSP, QC, mEPSP freq)
1b,c,d	*w^{1118}*	0.976 (0.032)	25.24 (1.073)	26.01 (0.976)	2.796 (0.121)	-67.243 (1.194)	8.241 (0.987)	17	
1b,c,d	*$hiw^{\Delta N}$*	0.457 (0.024)	6.471 (0.804)	13.890 (1.321)	1.33 (0.106)	-60.083 (1.256)	12.433 (1.876)	15	<0.0001 (****), <0.0001 (****), <0.0001 (****), <0.0001 (****)
1b,c,d	*c380-Gal4/+*	0.96 (0.073)	25.06 (1.234)	26.91 (2.11)	2.618 (0.194)	-69.65 (1.214)	9.765 (0.544)	8	0.9987 (ns), >0.9999 (ns), 0.9938 (ns), 0.9231 (ns)
1b,c,d	*c380-Gal4/+;UAS-wnd/+*	0.538 (0.016)	13.15 (0.794)	25.46 (1.549)	1.504 (0.105)	-60.140 (1.202)	12.109 (0.879)	17	<0.0001 (****), <0.0001 (****), 0.9979 (ns), <0.0001 (****)
1b,c,d	*$hiw^{\Delta N};wnd^3/wnd^{Df}$*	0.908 (0.048)	13.950 (0.759)	15.66 (1.071)	1.984 (0.221)	-65.066 (1.550)	7.487 (0.699)	10	0.6581 (ns), <0.0001 (****), <0.0001 (****) 0.0014 (**)

Figure	Genotype	RU486	mEPSP (mV)	EPSP (mV)	QC	mEPSP (Hz)	Resting Potential (mV)	Input Resistance (MΩ)	n	P value (significance) (mEPSP amp, EPSP, QC, mEPSP freq)
2e	*w^{1118}*	-	0.948 (0.047)	24.206 (1.564)	25.659 (1.387)	3.020 (0.155)	-69.564 (1.539)	8.764 (0.675)	8	
2e	*UAS-wnd/+;elav-GS/+*	- (24 hr)	0.859 (0.034)	21.777 (1.568)	25.508 (1.649)	2.987 (0.197)	-68.282 (1.923)	7.915 (0.445)	10	0.311 (ns), 0.8492 (ns), >0.9999 (ns), 0.8117 (ns)
2e	*UAS-wnd/+;elav-GS/+*	+ (24 hr)	0.603 (0.015)	13.9 (1.125)	23.079 (1.821)	1.678 (0.116)	-60.628 (1.228)	8.017 (0.666)	12	<0.0001 (****), 0.0002 (***), 0.8908 (ns), <0.0001 (****)
2e	*UAS-wnd/+;elav-GS/+*	- (48 hr)	0.837 (0.021)	22.012 (0.784)	26.5 (1.374)	2.881 (0.199)	-65.713 (0.783)	7.112 (0.368)	9	0.1271 (ns), 0.9109 (ns), >0.9999 (ns), 0.9115 (ns)
2e	*UAS-wnd/+;elav-GS/+*	+ (48 hr)	0.505 (0.023)	12.698 (0.738)	25.762 (2.147)	1.755 (0.132)	-57.522 (1.807)	6.998 (0.566)	10	<0.0001(****),<0.0001(****), 0.9998(ns), 0.0005 (***)
2e	*UAS-wnd/+;elav-GS/+*	- (72 hr)	0.913 (0.031)	24.225 (1.936)	26.684 (2.187)	3.232 (0.276)	-68.266 (2.618)	8.818 (0.506)	7	0.9857 (ns), >0.9999 (ns), 0.9998 (ns), 0.9997 (ns)
2e	*UAS-wnd/+;elav-GS/+*	+ (72 hr)	0.502 (0.023)	12.372 (1.313)	24.506 (2.056)	1.987 (0.244)	-58.887 (1.805)	8.335 (0.761)	9	<0.0001 (****), <0.0001 (****), 0.9758 (ns), 0.0045 (**)

Figure	Genotype	mEPSP (mV)	mEPSP (Hz)	n	P value (significance) (mEPSP amp, mEPSP freq)
3b	*w^{1118}*	0.914 (0.097)	2.962 (0.102)	5	
3b	*w^{1118};OK319-Gal4/+;UAS-TNT/+*	0.858 (0.039)	2.254 (0.111)	9	0.9087 (ns), 0.0001 (***)
3b	*$hiw^{\Delta N}$*	0.421 (0.038)	1.139 (0.08)	6	<0.0001 (****), <0.0001 (****)
3b	*$hiw^{\Delta N}$;OK319-Gal4/+; UAS-TNT/+*	0.335 (0.019)	0.662 (0.098)	7	<0.0001 (****),<0.0001 (****)
3b	*w^{1118};UAS-wnd/OK319-Gal4;UAS-TNT/+*	0.517 (0.024)	1.051 (0.026)	7	<0.0001 (****),<0.0001 (****)

Figure	Genotype	PhTx	mEPSP (mV)	EPSP (mV)	QC	mEPSP (Hz)	Resting Potential (mV)	Input Resistance (MΩ)	n	P value (significance) (mEPSP amp, EPSP, QC, mEPSP freq)
4b,c,d	*w^{1118}*	-	0.977 (0.019)	23.061 (0.671)	24.059 (0.951)	2.809 (0.091)	-65.029 (0.815)	8.235 (0.754)	25	
4b,c,d	*w^{1118}*	+	0.539 (0.021)	21.484 (0.763)	40.481 (2.115)	2.404 (0.123)	-67.881 (1.434)	9.110 (0.682)	17	<0.0001 (****), 0.9002 (ns), <0.0001 (****), 0.1407 (ns)
4b,c,d	*$hiw^{\Delta N}$*	-	0.442 (0.021)	6.303 (0.569)	14.780 (1.616)	1.288 (0.221)	-61.922 (0.981)	10.209 (0.555)	11	
4b,c,d	*$hiw^{\Delta N}$*	+	0.293 (0.008)	2.437 (0.337)	8.427 (1.243)	1.193 (0.114)	-66.853 (2.447)	10.027 (1.006)	10	<0.0001 (****), <0.0001 (****), 0.0059 (**), 0.9998 (ns)
4b,c,d	*c380-Gal4/+;UAS-wnd/+*	-	0.520 (0.032)	12.170 (1.316)	24.650 (2.816)	1.455 (0.141)	-61.485 (0.967)	9.115 (0.494)	7	
4b,c,d	*c380-Gal4/+;UAS-wnd/+*	+	0.372 (0.016)	6.172 (0.769)	16.960 (2.383)	1.212 (0.161)	-65.213 (2.327)	7.992 (0.507)	8	0.0029(**), 0.0022(**), 0.0587(ns), 0.9722 (ns)
4b,c,d	*$hiw^{\Delta N}$;wnd^{3}/wnd^{Df}*	-	0.938 (0.103)	16.572 (1.298)	18.271 (2.041)	1.997 (0.131)	-64.834 (2.141)	7.889 (0.658)	11	
4b,c,d	*$hiw^{\Delta N}$;wnd^{3}/wnd^{Df}*	+	0.487 (0.031)	13.326 (0.665)	27.544 (1.972)	1.597 (0.113)	-69.562 (2.623)	8.335 (0.761)	9	<0.0001 (****), 0.5174 (ns), 0.0043 (**), 0.5425 (ns)

Figure	Genotype	mEPSP (mV)	EPSP (mV)	QC	mEPSP (Hz)	Resting Potential (mV)	Input Resistance (MΩ)	n	P value (significance) (mEPSP amp, EPSP, QC, mEPSP freq)
5b,c,d	*w^{1118}*	0.947 (0.042)	23.944 (1.273)	26.911 (1.652)	3.003 (0.153)	-68.073 (1.791)	8.117 (0.659)	14	
5b,c,d	*w^{1118}; $GluRIIA^{sp16}$*	0.481 (0.020)	19.761 (0.981)	41.400 (2.939)	2.474 (0.093)	-69.291 (2.471)	9.175 (0.692)	8	<0.0001 (****), 0.2873 (ns), 0.0003 (***), 0.0829 (ns)
5b,c,d	*$hiw^{\Delta N}$*	0.455 (0.019)	7.463 (0.592)	16.620 (1.390)	1.45 (0.123)	-61.907 (0.982)	9.124 (0.805)	15	
5b,c,d	*$hiw^{\Delta N}$;$GluRIIA^{sp16}$*	0.273 (0.019)	1.601 (0.255)	5.792 (0.791)	1.492 (0.141)	-52.262 (0.711)	14.233 (1.236)	13	<0.0001 (****), 0.0244 (*), 0.0106 (*), 0.9960 (ns)

Figure	Genotype	PhTx	mEPSP (mV)	EPSP (mV)	QC	mEPSP (Hz)	Resting Potential (mV)	Input Resistance (MΩ)	n	P value (significance) (mEPSP amp, EPSP, QC, mEPSP freq)
6b,c,d,e	*w^{1118}*	-	1.022 (0.036)	22.93 (1.169)	22.84 (1.626)	2.954 (0.214)	-65.33 (1.7)	9.273 (0.768)	11	
6b,c,d,e	*w^{1118}; MHC-Gal4/UAS-Tor*	-	0.98 (0.075)	32.44 (1.98)	34.66 (3.717)	2.718 (0.139)	-64.86 (2.33)	10.361 (1.085)	9	0.9919 (ns), <0.0001 (****), 0.0083 (**), 0.9087 (ns)
6b,c,d,e	*w^{1118}; MHC-Gal4/UAS-Tor*	+	0.576 (0.013)	21.59 (1.559)	37.82 (3.317)	2.081 (0.132)	-67.578 (2.33)	8.336 (0.745)	9	<0.0001 (****), <0.0001 (****), 0.9544 (ns), 0.1533 (ns) (baseline is –PhTx)
6b,c,d,e	*$hiw^{\Delta N}$*	-	0.506 (0.02)	7.783 (0.668)	15.41 (1.155)	1.343 (0.142)	-58.67 (1.18)	8.909 (0.874)	13	
6b,c,d,e	*$hiw^{\Delta N}$; MHC-Gal4/UAS-Tor*	-	0.656 (0.03)	16.32 (0.674)	25.98 (1.731)	1.418 (0.111)	-60.87 (1.85)	13.769 (1.211)	16	0.0402 (*), <0.0001 (****), 0.0037 (**), 0.9988 (ns)
6b,c,d,e	*$hiw^{\Delta N}$; MHC-Gal4/UAS-Tor*	+	0.276 (0.03)	6.542 (0.786)	24.18 (3.212)	1.431 (0.224)	-55.195 (1.613)	11.54 (1.32)	6	<0.0001 (****), <0.0001 (****), 0.9954 (ns), >0.9999 (ns) (baseline is –PhTx)

Figure	Genotype	PhTx	mEPSP (mV)	EPSP (mV)	QC	mEPSP (Hz)	Resting Potential (mV)	Input Resistance (MΩ)	n	P value (significance) (mEPSP amp, EPSP, QC, mEPSP freq)
7d,e,f,g	*w^{1118}*	-	1.022 (0.036)	22.930 (1.169)	22.840 (1.626)	2.954 (0.214)	-65.327 (1.701)	7.914 (0.691)	11	
7d,e,f,g	*w^{1118}; MHC-Gal4/UAS-dGluRIIA*	-	1.398 (0.066)	31.360 (0.868)	22.75 (1.085)	2.813 (0.134)	-66.837 (1.507)	9.239 (0.992)	8	<0.0001 (****), <0.0001 (****), >0.9999 (ns), 0.9952 (ns)
7d,e,f,g	*w^{1118}; MHC-Gal4/UAS-dGluRIIA*	+	0.513 (0.031)	34.618 (1.903)	68.064 (4.763)	2.377 (0.275)	-65.642 (1.619)	9.239 (0.992)	8	<0.0001 (****), 0.0076 (**), <0.0001 (****), 0.7016 (ns) (baseline is –PhTx)
7d,e,f,g	*$hiw^{\Delta N}$*	-	0.506 (0.022)	7.783 (0.668)	15.410 (1.155)	1.343 (0.142)	-58.672 (1.141)	9.117 (0.867)	13	
7d,e,f,g	*$hiw^{\Delta N}$; MHC-Gal4/UAS-dGluRIIA*	-	0.735 (0.019)	14.800 (1.242)	20.350 (1.820)	1.294 (0.157)	-61.398 (1.028)	14.109 (1.299)	13	<0.0001 (****), <0.0001 (****), 0.4781 (ns), >0.9999 (ns)
7d,e,f,g	*$hiw^{\Delta N}$; MHC-Gal4/UAS-dGluRIIA*	+	0.400 (0.015)	6.703 (1.795)	18.680 (4.774)	1.111 (0.097)	-65.666 (3.798)	11.453 (0.916)	6	<0.0001 (****), 0.0015 (**), 0.9965 (ns), 0.9820 (ns) (baseline is –PhTx)

Figure	Genotype	mEPSP (mV)	EPSP (mV)	QC	mEPSP (Hz)	Resting Potential (mV)	Input Resistance (MΩ)	n	P value (significance) (mEPSP amp, EPSP, QC, mEPSP freq)
8b,c,d	w^{1118} (muscle 7)	0.966 (0.013)	24.080 (1.016)	24.920 (0.926)	3.026 (0.194)	-68.231 (3.061)	7.559 (0.661)	8	
8b,c,d	w^{1118}; *tubP>stop>Gal4, UAS-FLP, UAS-CD8-GFP/UAS-fasII-PEST+*; *H94-Gal4, nSyb-Gal80* (muscle 7)	1.296 (0.064)	22.840 (0.638)	17.810 (0.663)	3.004 (0.098)	-69.476 (3.312)	9.552 (0.891)	8	0.0096 (**), 0.3215 (ns), 0.0041 (**), 0.9207 (ns)

Figure	Genotype	mEPSP (mV)	EPSP (mV)	QC	mEPSP (Hz)	Resting Potential (mV)	Input Resistance (MΩ)	n	P value (significance) (mEPSP amp, EPSP, QC, mEPSP freq)
9b,c,d	$hiw^{\Delta N}$ (muscle 7)	0.446 (0.020)	8.405 (0.617)	18.790 (0.855)	1.605 (0.079)	-62.579 (2.904)	13.244 (1.276)	8	
9b,c,d	$hiw^{\Delta N}$; *tubP>stop>Gal4, UAS-FLP, UAS-CD8-GFP/UAS-fasII-PEST+*; *H94-Gal4, nSyb-Gal80* (muscle 7)	0.656 (0.037)	8.549 (0.665)	12.690 (0.499)	1.308 (0.113)	-62.195 (2.822)	14.559 (1.109)	6	0.0025 (**), 0.4848 (ns), 0.0248 (*), 0.0594 (ns)

Figure	Genotype	mEPSP (mV)	EPSP (mV)	QC	mEPSP (Hz)	Resting Potential (mV)	Input Resistance (MΩ)	n	P value (significance) (mEPSP amp, EPSP, QC, mEPSP freq)
S3b	w^{1118}	0.921 (0.019)	24.930 (1.242)	26.440 (1.038)	3.011 (0.098)	-67.243 (1.194)	8.241 (0.987)	7	
S3b	$hiw^{\Delta N}$;*OK6-Gal4/UAS-hiw*	0.845 (0.070)	20.560 (1.612)	13.890 (1.321)	2.959 (0.181)	-60.083 (1.256)	12.433 (1.876)	8	0.6206 (ns), 0.1808 (ns), 0.8825 (ns), 0.9935 (ns)
S3b	$hiw^{\Delta N}$;*UAS-hiw/+;MHC-Gal4/+*	0.505 (0.018)	9.164 (0.714)	22.040 (1.320)	1.153 (0.072)	-60.140 (1.202)	12.109 (0.879)	7	<0.0001 (****), <0.0001 (****), 0.0238 (*), <0.0001 (ns)
S3b	w^{1118};*UAS-wnd/+;MHC-Gal4/+*	0.878 (0.036)	26.970 (2.026)	15.960 (1.541)	3.193 (0.168)	-65.066 (1.550)	7.487 (0.699)	7	0.9709 (ns), 0.7798 (ns), 0.5345 (ns), 0.8101 (ns)

Figure	Genotype	RU486	PhTx	mEPSP (mV)	EPSP (mV)	QC	mEPSP (Hz)	Resting Potential (mV)	Input Resistance (MΩ)	n	P value (significance) (mEPSP amp, EPSP, QC, mEPSP freq)
S4b,c,d	w^{1118}	-	-	0.977 (0.019)	23.061 (0.671)	24.059 (0.951)	2.809 (0.091)	-65.029 (0.815)	8.235 (0.754)	25	
S4b,c,d	w^{1118}	-	+	0.539 (0.021)	21.484 (0.763)	40.481 (2.115)	2.404 (0.123)	-67.881 (1.434)	9.110 (0.682)	17	<0.0001 (****), 0.9002 (ns), <0.0001 (****), 0.1407 (ns)

S4b,c,d	*UAS-wnd/+;elav-GS/+*	- (48 hr)	-	0.8375 (0.021)	22.01 (0.784)	26.5 (1.374)	2.881 (0.199)	-65.713 (0.783)	7.112 (0.368)	9	
S4b,c,d	*UAS-wnd/+;elav-GS/+*	- (48hr)	+	0.4616 (0.029)	23.53 (2.35)	50.63 (3.811)	2.512 (0.223)	-65.257 (1.02)	8.282 (0.522)	9	<0.0001 (****), 0.8515 (ns), <0.0001 (****), 0.1716 (ns)
S4b,c,d	*UAS-wnd/+;elav-GS/+*	+ (48 hr)	-	0.505 (0.023)	12.7 (0.738)	24.76 (2.147)	1.755 (0.132)	-57.522 (1.807)	6.998 (0.566)	10	
S4b,c,d	*UAS-wnd/+;elav-GS/+*	+ (48 hr)	+	0.3372 (0.01)	6.901 (0.738)	20.81 (2.514)	1.512 (0.252)	-58.807 (1.135)	9.662 (0.691)	9	<0.0001 (****), <0.0001(****), 0.5289(ns), 0.9816 (ns)

Figure	Genotype	GluRIIA puncta sum intensity (%WT)	GluRIIB puncta sum intensity (%WT)	GluRIID puncta sum intensity (%WT)	n	P value (significance) (GluRIIA, GluRIIB, GluRIID)
1e-h	w^{1118}	100 (7.607)	100 (5.823)	100 (4.976)	14	
1e-h	$hiw^{\Delta N}$	26.390 (3.197)	46.966 (3.501)	18.824 (1.465)	13	<0.0001 (****), <0.0001 (****), <0.0001 (****)
1e-h	*c380-Gal4/+;UAS-wnd/+*	39.146 (4.497)	39.688 (2.916)	15.918 (0.996)	9	<0.0001 (****), <0.0001 (****), <0.0001 (****)
1e-h	$hiw^{\Delta N};wnd^{3}/wnd^{Df}$	85.547 (5.168)	108.629 (5.487)	110.211 (7.435)	9	0.2972 (ns), 0.5739 (ns), 0.4918 (ns)

Figure	Genotype	Dlg intensity (% wild type)	n	P value (significance)
1i	w^{1118}	100 (4.068)	10	
1i	$hiw^{\Delta N}$	39.311 (3.380)	9	<0.0001 (****)
1i	*c380-Gal4/+;UAS-wnd/+*	38.688 (4.406)	9	<0.0001 (****)
1i	$hiw^{\Delta N};wnd^{3}/wnd^{Df}$	112.393 (7.456)	11	0.6416 (ns)

Figure	Genotype	RU486	bouton #/NMJ	neuronal surface area (μm^2)	vGlut intensity (% wild type)	n	P value (significance) (bouton #, area, vGlut)
2c	w^{1118}	-	42.270 (2.913)	285.939 (20.173)	100 (7.768)	14	
2c	*UAS-wnd/+;elav-GS/+*	- (24 hr)	36.888 (3.529)	240.207 (22.266)	95.766 (6.267)	9	0.7729 (ns), 0.9778 (ns), >0.9999 (ns)
2c	*UAS-wnd/+;elav-GS/+*	+ (24 hr)	38.818 (3.516)	228.818 (95.34)	94.191 (4.059)	11	0.7059 (ns), 0.7334 (ns), 0.3202 (ns)
2c	*UAS-wnd/+;elav-GS/+*	- (48 hr)	31.363 (1.46)	202.054 (14.747)	97.887 (5.298)	11	0.5449 (ns), 0.2229 (ns), 0.9997 (ns)

2c	*UAS-wnd/+;elav-GS/+*	+ (48 hr)	35.083 (3.066)	189.583 (18.74)	65.234 (2.215)	12	0.2998 (ns), 0.6109 (ns),<0.0001 (****)
2c	*UAS-wnd/+;elav-GS/+*	- (72 hr)	35.545 (2.8)	182.009 (13.687)	93.911 (7.118)	11	0.6839 (*), 0.0319 (*), 0.7107 (ns)
2c	*UAS-wnd/+;elav-GS/+*	+ (72 hr)	50.11 (4.137)	276.287 (27.259)	64.941 (3.791)	10	0.0097 (**), 0.0064 (**), <0.0001 (****)

Figure	Genotype	RU486	GluRIIA #/NMJ	GluRIIA density (#/μm^2)	GluRIIB #/NMJ	GluRIIB density (#/μm^2)	n	P value (significance) (IIA #, IIA density, IIB #, IIB density)
2g	w^{1118}	-	114.103 (10.211)	0.411 (0.029)	122.016 (10.198)	0.428 (0.311)	14	
2g	*UAS-wnd/+;elav-GS/+*	- (24 hr)	109.555 (8.032)	0.456 (0.036)	114.11 (12.278)	0.475 (0.058)	9	0.3969 (ns), 0.6798 (ns), 0.9996 (ns), 0.5512 (ns)
2g	*UAS-wnd/+;elav-GS/+*	+ (24 hr)	128.363 (11.536)	0.562 (0.051)	126.272 (9.856)	0.554 (0.043)	11	0.2278 (ns), 0.1195 (ns), 0.4714 (ns), 0.2822 (ns)
2g	*UAS-wnd/+;elav-GS/+*	- (48 hr)	118.363 (4.258)	0.558 (0.02)	115.363 (4.525)	0.519 (0.02)	11	0.9879 (ns), 0.0929 (ns), 0.8917 (ns), 0.2978 (ns)
2g	*UAS-wnd/+;elav-GS/+*	+ (48 hr)	107.417 (7.644)	0.537 (0.038)	100 (6.574)	0.5 (0.032)	12	0.29360 (ns), 0.6371 (ns),0.0725 (ns), 0.6242 (ns)
2g	*UAS-wnd/+;elav-GS/+*	- (72 hr)	103.272 (7.214)	0.543 (0.037)	96.545 (5.365)	0.508 (0.028)	11	0.7819 (ns), 0.1329 (ns), 0.1989 (ns), 0.2667 (ns)
2g	*UAS-wnd/+;elav-GS/+*	+ (72 hr)	124.7 (7.279)	0.452 (0.026)	119.4 (9.182)	0.433 (0.033)	10	0.0554 (ns), 0.0701 (ns), 0.0469 (*), 0.1061 (ns)

Figure	Genotype	RU486	GluRIIA puncta mean intensity (% WT)	GluRIIA puncta area (μm^2)	GluRIIA puncta sum intensity (% WT)	n	P value (significance) (IIA mean, IIA area, IIA sum)
2g	w^{1118}	-	100 (7.201)	0.973 (0.053)	100 (9.108)	14	
2g	*UAS-wnd/+;elav-GS/+*	- (24 hr)	91.776 (2.173)	1.197 (0.068)	104.11 (9.097)	9	0.5449 (ns), 0.4221 (ns), 0.9998 (ns),
2g	*UAS-wnd/+;elav-GS/+*	+ (24 hr)	78.352 (1.873)	0.522 (0.026)	33.845 (1.736)	11	<0.0001 (****),<0.0001 (****), <0.0001 (****)
2g	*UAS-wnd/+;elav-GS/+*	- (48 hr)	94.556 (3.827)	0.909 (0.049)	93.228 (3.597)	11	0.6319 (ns), 0.5512 (ns), 0.5417 (ns)
2g	*UAS-wnd/+;elav-GS/+*	+ (48 hr)	74.197 (2.385)	0.405 (0.027)	38.498 (2.715)	12	<0.0001 (****),<0.0001 (****), <0.0001 (****)
2g	*UAS-wnd/+;elav-GS/+*	- (72 hr)	101.211 (5.213)	1.002 (0.078)	104.554 (7.229)	11	0.9819 (ns), 0.9229 (ns), 0.7995 (ns)
2g	*UAS-wnd/+;elav-GS/+*	+ (72 hr)	79.888 (4.614)	0.535 (0.047)	41.612 (3.167)	10	0.0074 (****),<0.0001 (****), <0.0001 (****)

Figure	Genotype	RU486	GluRIIB puncta mean intensity (% WT)	GluRIIB puncta area (μm^2)	GluRIIB puncta sum intensity (% WT)	n	P value (significance) (IIA mean, IIA area, IIA sum)
2g	*w^{1118}*	-	100 (8.211)	0.965 (0.142)	100 (9.918)	14	
2g	*UAS-wnd/+;elav-GS/+*	- (24 hr)	95.172 (4.827)	0.887 (0.046)	94.091 (7.109)	9	0.6429 (ns), 0.3211 (ns), 0.2971 (ns),
2g	*UAS-wnd/+;elav-GS/+*	+ (24 hr)	99.365 (2.935)	0.887 (0.045)	105.805 (7.115)	11	0.9081 (ns), 0.9927 (ns), 0.5549 (ns)
2g	*UAS-wnd/+;elav-GS/+*	- (48 hr)	102.515 (4.817)	0.971 (0.051)	99.213 (4.521)	11	0.9969 (ns), 0.9592 (ns), 0.9998 (ns)
2g	*UAS-wnd/+;elav-GS/+*	+ (48 hr)	69.394 (1.927)	0.652 (0.03)	55.803 (2.973)	12	<0.0001 (****),<0.0001 (****), 0.0007 (***)
2g	*UAS-wnd/+;elav-GS/+*	- (72 hr)	96.998 (5.119)	1.048 (0.075)	101.584 (7.116)	11	0.7809 (ns), 0.5203 (ns), 0.9916 (ns)
2g	*UAS-wnd/+;elav-GS/+*	+ (72 hr)	58.729 (6.918)	0.728 (0.079)	38.232 (3.286)	10	0.0070 (***), 0.0101 (*), 0.0002 (***)

Figure	Genotype	bouton #/NMJ	GluRIIC puncta sum intensity (% wild type)	GluRIID puncta sum intensity (% wild type)	n (bouton, IIC-IID)	P value (significance) (bouton #, GluRIIC, GluRIID)
3d,f	*w^{1118}*	44.270 (3.003)	100 (4.742)	100 (5.768)	11, 8	
3d,f	*w^{1118};OK319-Gal4/+;UAS-TNT/+*	43.750 (2.845)	129.743 (5.955)	133.594 (8.921)	12, 9	>0.9999 (ns), 0.0054 (**), 0.0028 (**)
3d,f	*$hiw^{\Delta N}$*	116.8 (9.785)	34.352 (5.631)	25.222 (2.522)	8, 6	<0.0001 (****), <0.0001 (****),<0.0001 (****)
3d,f	*$hiw^{\Delta N}$;OK319-Gal4/+; UAS-TNT/+*	92.000 (8.191)	44.788 (5.631)	37.036 (3.262)	7, 7	<0.0001 (****),<0.0001 (****)
3d,f	*w^{1118};UAS-wnd/OK319-Gal4;UAS-TNT/+*	70.220 (4.193)	49.026 (4.722)	47.119 (3.799)	9, 6	<0.0066 (**), <0.0001 (****),<0.0001 (****)

Figure	Genotype	GluRIIA puncta sum intensity (% wild type)	GluRIIB puncta sum intensity (% wild type)	GluRIID puncta sum intensity (% wild type)	Dlg mean intensity (% wild type)	n	P value (significance) (GluRIIA, GluRIIB, GluRIID, Dlg)
6g	*w^{1118}*	100 (9.628)	100 (7.887)	100 (10.910)	100 (5.643)	12	
6g	*w^{1118}; MHC-Gal4/UAS-Tor*	96.201 (6.556)	101.123 (9.109)	110.625 (8.924)	129.006 (4.566)	11	0.8896 (ns), 0.9998 (ns), 0.9073 (ns), 0.0005 (***)
6g	*$hiw^{\Delta N}$*	100 (5.211)	100 (7.358)	100 (10.330)	100 (4.982)	7	
6g	*$hiw^{\Delta N}$; MHC-Gal4/UAS-Tor*	209.72 (14.53)	171.27 (14.78)	248.98 (23.37)	122.098 (2.02)	8	0.0004 (***), 0.0034 (**), <0.0001 (****), 0.0008 (***)

Figure	Genotype	GluRIIA puncta #/NMJ	GluRIIB puncta #/NMJ	GluRIID puncta #/NMJ	n	P value (significance) (GluRIIA, GluRIIB, GluRIID)
6g	w^{1118}	122.133 (10.315)	112.926 (8.514)	116.098 (7.908)	12	
6g	w^{1118}; *MHC-Gal4/UAS-Tor*	115.502 (8.228)	108.639 (7.227)	120.472 (9.105)	11	0.8106 (ns), 0.9711 (ns), 0.8872 (ns)
6g	$hiw^{\Delta N}$	88.5 (6.951)	95.714 (10.228)	94.5 (8.101)	7	
6g	$hiw^{\Delta N}$; *MHC-Gal4/UAS-Tor*	163.375 (10.121)	187.25 (11.597)	81.607 (7.987)	8	<0.0001 (****), <0.0001 (****), 0.0022 (***)

Figure	Genotype	GluRIIA density (#/μm^2)	GluRIIB density (#/μm^2)	GluRIID density (#/μm^2)	n	P value (significance) (GluRIIA, GluRIIB, GluRIID)
6g	w^{1118}	0.464 (0.039)	0.465 (0.042)	0.473 (0.031)	12	
6g	w^{1118}; *MHC-Gal4/UAS-Tor*	0.511 (0.028)	0.459 (0.031)	0.481 (0.022)	11	0.6116 (ns), 0.9181 (ns), 0.8709 (ns)
6g	$hiw^{\Delta N}$	0.261 (0.016)	0.279 (0.019)	0.219 (0.018)	7	
6g	$hiw^{\Delta N}$; *MHC-Gal4/UAS-Tor*	0.429 (0.026)	0.493 (0.03)	0.401 (0.042)	8	<0.0001 (****), <0.0001 (****),, 0.0007 (***)

Figure	Genotype	GluRIIA puncta mean intesnity (%WT)	GluRIIB puncta mean intesnity (%WT)	GluRIID puncta mean intesnity (%WT)	n	P value (significance) (GluRIIA, GluRIIB, GluRIID)
6g	w^{1118}	100 (8.129)	100 (9.116)	100 (11.008)	12	
6g	w^{1118}; *MHC-Gal4/UAS-Tor*	97.271 (9.104)	109.093 (8.954)	105.995 (10.098)	11	0.7015 (ns), 0.6911 (ns), 0.6952 (ns)
6g	$hiw^{\Delta N}$	100 (5.136)	100 (7.123)	100 (9.728)	7	
6g	$hiw^{\Delta N}$; *MHC-Gal4/UAS-Tor*	149.712 (12.193)	140.097 (14.081)	179.238 (15.455)	8	0.0031 (**), 0.0078 (**), 0.009 (***)

Figure	Genotype	GluRIIA puncta area (μm^2)	GluRIIB puncta area (μm^2)	GluRIID puncta area (μm^2)	n	P value (significance)<br>(GluRIIA, GluRIIB, GluRIID)
6g	*w^{1118}*	1.013<br>(0.043)	0.982<br>(0.076)	1.111<br>(0.053)	12	
6g	*w^{1118}; MHC-Gal4/UAS-Tor*	0.998<br>(0.029)	0.976<br>(0.063)	0.951<br>(0.0487)	11	0.7412 (ns), 0.7617 (ns), 0.5448 (ns)
6g	*$hiw^{\Delta N}$*	0.409<br>(0.014)	0.439<br>(0.028)	0.499<br>(0.032)	7	
6g	*$hiw^{\Delta N}$; MHC-Gal4/UAS-Tor*	0.774<br>(0.028)	0.808<br>(0.049)	0.81<br>(0.057)	8	<0.0001 (****), 0.001 (**), 0.0017 (**)

Figure	Genotype	GluRIIA puncta sum intensity (% wild type)	GluRIIB puncta sum intensity (% wild type)	GluRIID puncta sum intensity (% wild type)	n	P value (significance)<br>(GluRIIA, GluRIIB, GluRIID)
7b	*w^{1118}*	100<br>(10.117)	100<br>(9.556)	100<br>(7.099)	12	
7b	*w^{1118}; MHC-Gal4/UAS-dGluRIIA*	96.201<br>(6.556)	101.123<br>(9.109)	110.625<br>(8.924)	10	0.0037 (**), <0.0001 (****), 0.7654 (ns)
7b	*$hiw^{\Delta N}$*	100<br>(9.091)	100<br>(6.821)	100<br>(10.398)	9	
7b	*$hiw^{\Delta N}$; MHC-Gal4/UAS-dGluRIIA*	243.231<br>(15.625)	136.151<br>(12.889)	277.455<br>(17.729)	8	0.0009 (***), 0.0355 (*), 0.0006 (***)

Figure	Genotype	bouton #/NMJ (muscle 4)	GluRIIA intensity (% wild type)	n (bouton #, GluRIIA intensity)	P value (significance)<br>(bouton #, vGlut intensity)
8f,g	*w^{1118}*<br>(muscle 7)	39.750<br>(1.509)	100<br>(10.142)	8, 8	
8f,g	*w^{1118}; tubP>stop>Gal4, UAS-FLP, UAS-CD8-GFP/UAS-fasII-PEST+; H94-Gal4, nSyb-Gal80*<br>(muscle 7)	19.710<br>(2.201)	198.320<br>(22.312)	7, 7	<0.0001 (****), 0.0035 (**)

Figure	Genotype	Bouton #/NMJ	GluRIIA intensity (% wild type)	n	P value (significance)<br>(Bouton #, GluRIIA intensity)
9f,g	*$hiw^{\Delta N}$*<br>(muscle 7)	55.140<br>(3.348)	33.831<br>(3.825)	7	
9f,g	*$hiw^{\Delta N}$; tubP>stop>Gal4, UAS-FLP, UAS-CD8-GFP/UAS-fasII-PEST+; H94-Gal4, nSyb-Gal80*<br>(muscle 7)	34.860<br>(4.405)	82.918<br>(12.628)	7	0.0036 (**), 0.0184 (*)

Figure	Genotype	neuronal surface area (μm^2)	vGlut puncta mean intensity (%WT)	BRP puncta density (#/μm^2)	BRP-GluRIII apposition	n	P value (significance) (neuronal area, vGlut intensity, density, apposition)
S1b,c,d,e	*w^{1118}*	295.939 (18.673)	100 (8.319)	1.064 (0.115)	0.859 (0.024)	14	
S1b,c,d,e	*$hiw^{\Delta N}$*	431.122 (21.326)	39.677 (3.788)	0.650 (0.062)	0.706 (0.031)	10	0.0006 (***),<0.0001 (****), 0.0101 (*), 0.0003 (***)
S1b,c,d,e	*c380-Gal4/+;UAS-wnd/+*	374.600 (25.521)	39.677 (3.788)	0.371 (0.031)	0.650 (0.037)	10	0.0199 (*), <0.0001 (****), <0.0001 (****), <0.0001 (****)
S1b,c,d,e	*$hiw^{\Delta N};wnd^3/wnd^{Df}$*	302.666 (11.491)	97.128 (9.906)	1.028 (0.085)	0.860 (0.017)	12	0.9354 (ns), 0.9867 (ns), 0.9909 (ns), >0.9999 (ns)

Figure	Genotype	GluRIIA puncta #/NMJ	GluRIIB puncta #/NMJ	GluRIID puncta #/NMJ	neuronal surface area (μm^2)	n	P value (significance) (GluRIIA, GluRIIB, GluRIID, neuronal area)
S2b,c,d,e	*w^{1118}*	134.153 (11.305)	142.076 (9.547)	136.692 (9.110)	288.796 (16.418)	14	
S2b,c,d,e	*$hiw^{\Delta N}$*	93.600 (6.586)	112.933 (10.548)	94.500 (8.101)	395.525 (19.568)	13	0.0056 (**), 0.1098 (ns), 0.0003 (***), 0.0006 (***)
S2b,c,d,e	*c380-Gal4/+;UAS-wnd/+*	106.375 (8.542)	106.375 (10.135)	106.750 (9.759)	364.6 (22.563)	9	0.1514 (ns), 0.0698 (ns), 0.0617 (****), 0.0199 (*)
S2b,c,d,e	*$hiw^{\Delta N};wnd^3/wnd^{Df}$*	126.125 (2.496)	124.375 (5.172)	123 (2.299)	302.666 (11.491)	9	0.9229 (ns), 0.5934 (ns), 0.6383 (ns), 0.9354 (ns)

Figure	Genotype	GluRIIA puncta density (μm^2)	GluRIIB puncta density (μm^2)	GluRIID puncta density (μm^2)	n	P value (significance) (GluRIIA, GluRIIB, GluRIID)
S2b,c,d,e	*w^{1118}*	0.464 (0.039)	0.465 (0.042)	0.473 (0.031)	14	
S2b,c,d,e	*$hiw^{\Delta N}$*	0.236 (0.016)	0.285 (0.026)	0.238 (0.020)	13	<0.0001 (****), <0.0001 (****), <0.0001 (****)
S2b,c,d,e	*c380-Gal4/+;UAS-wnd/+*	0.292 (0.023)	0.292 (0.027)	0.293 (0.026)	9	0.0009 (***), 0.0002 (***), <0.0001 (****)
S2b,c,d,e	*$hiw^{\Delta N};wnd^3/wnd^{Df}$*	0.417 (0.008)	0.411 (0.017)	0.406 (0.007)	9	0.6541 (ns), 0.2345 (ns), 0.2496 (ns)

Figure	Genotype	GluRIIA puncta area (μm^2)	GluRIIB puncta area (μm^2)	GluRIID puncta area (μm^2)	n	P value (significance) (GluRIIA, GluRIIB, GluRIID)
S2b,c,d,e	*w^{1118}*	0.973 (0.053)	0.969 (0.052)	1.182 (0.046)	14	
S2b,c,d,e	*$hiw^{\Delta N}$*	0.417 (0.034)	0.454 (0.039)	0.507 (0.035)	13	<0.0001 (****), <0.0001 (****), <0.0001 (****)

S2b,c,d,e	*c380-Gal4/+;UAS-wnd/+*	0.577 (0.042)	0.513 (0.056)	0.545 (0.039)	9	0.0008 (***), <0.0001 (****), <0.0001 (****)
S2b,c,d,e	*hiw$^{\Delta N}$;wnd^{3}/wnd^{Df}*	0.956 (0.114)	1.036 (0.074)	1.09 (0.082)	9	0.9976 (ns), 0.8270 (ns), 0.4679 (ns)

Figure	Genotype	GluRIIA puncta mean intensity (%WT)	GluRIIB puncta mean intensity (%WT)	GluRIID puncta mean intensity (%WT)	n	P value (significance) (GluRIIA, GluRIIB, GluRIID)
S2b,c,d,e	*w^{1118}*	100 (4.222)	100 (3.352)	100 (2.671)	14	
S2b,c,d,e	*hiw$^{\Delta N}$*	44.386 (3.090)	61.151 (3.934)	27.635 (1.574)	13	<0.0001 (****), <0.0001 (****), <0.0001 (****)
S2b,c,d,e	*c380-Gal4/+;UAS-wnd/+*	42.721 (3.513)	51.295 (3.370)	36.931 (2.353)	9	<0.0001 (****), <0.0001 (****), <0.0001 (****)
S2b,c,d,e	*hiw$^{\Delta N}$;wnd^{3}/wnd^{Df}*	95.495 (5.290)	108.543 (2.878)	99.762 (3.676)	9	0.8687 (ns), 0.3669 (ns), >0.9999 (ns)

Figure	Genotype	bouton #/NMJ (muscle 4)	vGlut puncta mean intensity (% wild type)	n (bouton #, vGlut intensity)	P value (significance) (bouton #, vGlut intensity)
S3d	*w^{1118}*	45.200 (2.936)	100 (2.558)	15,10	
S3d	*hiw$^{\Delta N}$;OK6-Gal4/UAS-hiw*	46.82 (3.739)	87.042 (5.849)	11,8	0.9813 (ns), 0.1394 (ns)
S3d	*hiw$^{\Delta N}$;UAS-hiw/+;MHC-Gal4/+*	84.850 (2.839)	66.337 (10.036)	13,8	<0.0001 (****), <0.0001 (****)
S3d	*w^{1118};UAS-wnd/+;MHC-Gal4/+*	42.780 (2.623)	112.011 (5.031)	9,10	0.9504 (ns), 0.1500 (ns)

Figure	Genotype	DLG intensity (% wild type)	GluRIIA intensity (% wild type)	GluRIIB intensity (% wild type)	GluRIID intensity (% wild type)	n (DLG, GluR)	P value (significance) (DLG, GluRIIA, GluRIIB, GluRIID)
S3f	*w^{1118}*	100 (7.393)	100 (5.043)	100 (8.816)	100 (4.096)	10,8	
S3f	*hiw$^{\Delta N}$;OK6-Gal4/UAS-hiw*	93.530 (6.512)	91.218 (11.946)	119.557 (9.491)	88.278 (3.571)	8,7	0.8743 (ns), 0.8308 (ns), 0.3296 (ns), 0.2916 (ns)
S3f	*hiw$^{\Delta N}$;UAS-hiw/+;MHC-Gal4/+*	44.329 (1.023)	33.207 (3.603)	43.466 (3.405)	20.113 (1.398)	8,7	<0.0001 (****), <0.0001 (****), 0.0002 (***), <0.0001 (****)
S3f	*w^{1118};UAS-wnd/+;MHC-Gal4/+*	95.447 (6.280)	108.040 (6.543)	107.218 (8.302)	102.613 (6.654)	9,8	0.9462 (ns), 0.8519 (ns), 0.1397 (ns), 0.9750 (ns)

Figure	Genotype	bouton #/NMJ (muscle 4)	vGlut puncta mean intensity (% wild type)	n (bouton #, vGlut intensity)	P value (significance) (bouton #, vGlut intensity)
S5b,c	*w^{1118}*	42.920 (3.524)	100 (3.066)	13,8	
S5b,c	*w^{1118}; MHC-Gal4/UAS-Tor*	39.230 (4.450)	112.971 (4.801)	13,8	0.9602 (ns), 0.9315 (ns)
S5b,c	*$hiw^{\Delta N}$*	107.100 (7.731)	66.893 (1.021)	13,8	<0.0001 (****), <0.0001 (****)
S5b,c	*$hiw^{\Delta N}$; MHC-Gal4/UAS-Tor*	87.200 (4.749)	73.224 (3.669)	10,8	0.0774 (ns), 0.6730 (ns)

Figure	Genotype	puc-lacZ intensity (% wild type)	n	P value (significance) (bouton #, vGlut intensity)
S5d	*w^{1118} ;puc-lacZ/+*	100 (7.169)	6	
S5d	*w^{1118}; G14-Gal4/UAS-Tor;puc-lacZ/+*	95.971 (8.801)	6	0.9932 (ns)
S5d	*$hiw^{\Delta N}$;puc-lacZ/+*	238.009 (17.866)	5	<0.0001 (****)
S5d	*$hiw^{\Delta N}$; G14-Gal4/UAS-Tor;puc-lacZ/+*	245.76 (21.45)	5	0.8861 (ns)

Figure	Genotype	neuronal surface area (μm^2)	Dlg mean intensity (%WT)	n	P value (significance) (neuronal area, DLG intensity)
S6b,c	*w^{1118}*	288.796 (16.418)	100 (6.119)	14	
S6b,c	*w^{1118}; MHC-Gal4/UAS-dGluRIIA*	276.333 (29.321)	127.684 (10.152)	10	0.9707 (ns), 0.0081 (**)
S6b,c	*$hiw^{\Delta N}$*	395.525 (18.655)	41.956 (4.459)	10	0.0021 (**), <0.0001 (****)
S6b,c	*$hiw^{\Delta N}$; MHC-Gal4/UAS-dGluRIIA*	422.186 (7.838)	76.188 (6.714)	12	0.0006 (***),<0.0001 (****)

Figure	Genotype	GluRIIA puncta #/NMJ	GluRIIB puncta #/NMJ	GluRIID puncta #/NMJ	n	P value (significance) (GluRIIA, GluRIIB, GluRIID)
S6d	*w^{1118}*	134.153 (11.305)	142.076 (9.547)	136.692 (9.110)	13	
S6d	*w^{1118}; MHC-Gal4/UAS-dGluRIIA*	124.111 (6.613)	15.25 (0.752)	124.75 (10.455)	9	0.8482 (ns), <0.0001 (****), 0.7722 (ns)

S6d	*hiw*$^{\Delta N}$	93.6 (6.586)	112.933 (10.548)	94.5 (8.101)	12	0.0056 (**), 0.0802 (ns), 0.0009 (***)
S6d	*hiw*$^{\Delta N}$; *MHC-Gal4/UAS-dGluRIIA*	151.143 (5.125)	116.044 (5.33)	140 (7.488)	7	0.5867 (ns), 0.2492 (ns), 0.9941 (ns)

**Figure**	**Genotype**	**GluRIIA density (#/μm^2)**	**GluRIIB puncta (#/μm^2)**	**GluRIID puncta (#/μm^2)**	**n**	**P value (significance) (GluRIIA, GluRIIB, GluRIID)**
S6e	*w*^{1118}	0.492 (0.051)	0.492 (0.033)	0.473 (0.031)	13	
S6e	*w*^{1118}; *MHC-Gal4/UAS-dGluRIIA*	0.476 (0.038)	0.055 (0.003)	0.451 (0.038)	9	0.9907 (ns), <0.0001 (****), 0.9476 (ns)
S6e	*hiw*$^{\Delta N}$	0.226 (0.023)	0.285 (0.027)	0.228 (0.017)	12	<0.0001 (****), <0.0001 (****), <0.0001 (****)
S6e	*hiw*$^{\Delta N}$; *MHC-Gal4/UAS-dGluRIIA*	0.369 (0.012)	0.283 (0.017)	0.342 (0.019)	7	0.1679 (ns), <0.0001 (****), 0.0176 (*)

**Figure**	**Genotype**	**GluRIIA puncta mean intesnity (%WT)**	**GluRIIB puncta mean intesnity (%WT)**	**GluRIID puncta mean intesnity (%WT)**	**n**	**P value (significance) (GluRIIA, GluRIIB, GluRIID)**
S6f	*w*^{1118}	100 (4.222)	100 (3.352)	100 (2.671)	13	
S6f	*w*^{1118}; *MHC-Gal4/UAS-dGluRIIA*	133.956 (3.091)	17.768 (0.808)	90.916 (1.898)	9	<0.0001 (****), <0.0001 (****), 0.1061 (ns)
S6f	*hiw*$^{\Delta N}$	44.384 (3.785)	61.151 (4.818)	49.744 (6.977)	12	<0.0001 (****), <0.0001 (****), <0.0001 (****)
S6f	*hiw*$^{\Delta N}$; *MHC-Gal4/UAS-dGluRIIA*	96.195 (2.438)	69.205 (1.711)	96.62 (2.004)	7	0.9120 (ns), <0.0001 (****), 0.8363 (ns)

**Figure**	**Genotype**	**GluRIIA puncta area (μm^2)**	**GluRIIB puncta area (μm^2)**	**GluRIID puncta area (μm^2)**	**n**	**P value (significance) (GluRIIA, GluRIIB, GluRIID)**
S6f	*w*^{1118}	0.973 (0.053)	0.969 (0.052)	1.182 (0.047)	13	
S6f	*w*^{1118}; *MHC-Gal4/UAS-dGluRIIA*	1.458 (0.153)	0.088 (0.004)	1.067 (0.067)	9	0.0024 (**), <0.0001 (****), 0.2632 (ns)
S6f	*hiw*$^{\Delta N}$	0.416 (0.032)	0.454 (0.048)	0.506 (0.035)	12	<0.0001 (****), <0.0001 (****), <0.0001 (****)
S6f	*hiw*$^{\Delta N}$; *MHC-Gal4/UAS-dGluRIIA*	0.952 (0.031)	0.644 (0.023)	1.011 (0.05)	7	0.9985 (ns), <0.0001 (****), 0.0704 (ns)
